# Supplementary material for: Development of a computer-aided design software for the quantitative evaluation of aesthetic damage
Source: PLoS One. 2019 Dec 18;14(12):e0226322. doi: 10.1371/journal.pone.0226322 (PMC6919621; doi:10.1371/journal.pone.0226322)
Supplement: S1 Code site — 1. (ZIP) [file pone.0226322.s001.zip › [EESC_jr][Projeto]Relatorio_07_21/reports/templates_zip/gentelella-master/vendors/morris.js/examples/goals.html]

# Value Goals

```
var decimal_data = [];
for (var x = 0; x <= 360; x += 10) {
  decimal_data.push({
    x: x,
    y: Math.sin(Math.PI * x / 180).toFixed(4)
  });
}
window.m = Morris.Line({
  element: 'graph',
  data: decimal_data,
  xkey: 'x',
  ykeys: ['y'],
  labels: ['sin(x)'],
  parseTime: false,
  goals: [-1, 0, 1]
});
```
